# Genetics affects choice of academic subjects as well as achievement

**DOI:** 10.1038/srep26373

**Published:** 2016-06-16

**Authors:** Kaili Rimfeld, Ziada Ayorech, Philip S. Dale, Yulia Kovas, Robert Plomin

**Affiliations:** 1 King’s College London, MRC Social, Genetic and Developmental Psychiatry Centre, Institute of Psychiatry, Psychology & Neuroscience, London, SE5 8AF, UK; 2 University of New Mexico, Department of Speech and Hearing Sciences, Albuquerque, NM, 87131, USA; 3 Goldsmiths, University of London, Department of Psychology, London, SE14 6NW, UK; 4 Tomsk State University, Laboratory for Cognitive Investigations and Behavioural Genetics, Tomsk, 634050, Russia

## Abstract

We have previously shown that individual differences in educational achievement are highly heritable throughout compulsory education. After completing compulsory education at age 16, students in England can choose to continue to study for two years (A-levels) in preparation for applying to university and they can freely choose which subjects to study. Here, for the first time, we show that choosing to do A-levels and the choice of subjects show substantial genetic influence, as does performance after two years studying the chosen subjects. Using a UK-representative sample of 6584 twin pairs, heritability estimates were 44% for choosing to do A-levels and 52–80% for choice of subject. Achievement after two years was also highly heritable (35–76%). The findings that DNA differences substantially affect differences in appetites as well as aptitudes suggest a genetic way of thinking about education in which individuals actively create their own educational experiences in part based on their genetic propensities.

Educational achievement is a strong predictor of many life outcomes, such as higher education, occupation, health and even life expectancy^1^,^2^,^3^. Because differences in children’s educational achievement and the subject choices they make in secondary school will propel young individuals on to a variety of lifelong trajectories, it is important to understand what influences the subject choices students take after compulsory education and to understand why students differ so widely in school grades. Subject choice after compulsory education is especially important as all academic learning after the age of 16 in England and Wales is considered to be preparation for further education and university entry.

Educational achievement has been studied using quantitative genetic methods, such as the classical twin method that compares identical twins to non-identical twins, to estimate the extent to which individual differences in school achievement are influenced by genetic factors and shared or non-shared environmental factors. Shared environmental factors that contribute to the similarities between siblings raised in the same family^4^, for example home or school environment, are certainly important, as children have to be taught skills such as reading and writing, they have to gain knowledge of scientific theories and historical facts, and they need guidance to appreciate music and art. Nonetheless, children from the same home, attending the same school and even the same classroom differ in academic performance, indicating that other factors besides shared environmental factors must be present. Previous research has shown that educational achievement is substantially heritable from the early school years until the end of compulsory education, which means that, to a large extent, differences in children’s educational achievement can be explained by inherited differences in children’s DNA sequence^5^,^6^,^7^,^8^,^9^. It is reasonable to assume that this high heritability of educational achievement is explained by children’s aptitude, or intelligence, but we have shown that educational achievement in the early school years is even more heritable than intelligence^10^. Furthermore, our recent studies have shown that the high heritability of educational achievement at the end of compulsory education is not explained by intelligence alone, but rather is influenced by a constellation of genetically related traits, such as self-efficacy, behavioral problems, and personality^11^,^12^.

Previous research demonstrates that genetic differences between children not only influence how well they perform at school, but also how easy or enjoyable they find learning in general^13^,^14^. It is also noteworthy that children may find certain subjects more enjoyable than others even when their achievement is good across subjects^11^,^14^. We hypothesize that given a choice, children will select, modify and create their own educational experiences in part based on their genetic propensities, a concept known as genotype-environment correlation^15^. These findings suggest that children are not passive recipients of instruction, but instead are active participants in their path to knowledge. In a more personalized education system, children would choose educational subjects early allowing them to focus on their strengths and interests. However, until the age of 16, students in England and Wales have little choice. At age 14 when they start their GCSE (General Certificate of Secondary Education) course, students are given some choice; however, English, mathematics and sciences are compulsory subjects for GCSE, and some schools require students to take separate science courses (biology, physics and chemistry), as compared to a combined science course. Many schools also require students to take English literature and at least one modern foreign language course, while others restrict them to only one foreign language course. Although students typically take 10 GCSE subjects, the differences in requirements across schools interferes with the investigation of student choices. For these reasons we were previously unable to investigate genetic influence on subject choice.

At age 16, after compulsory education, it is possible to study choice. Students can choose to study towards the A-levels (General Certificate of Education Advanced Level), a two-year course, which is a prerequisite for higher education. For the first time in their educational experience, students are free to choose all of their A-level subjects from over 80 different options, typically choosing three to four A-level courses. However, despite the importance of choosing to do A-levels and subject choices, it is largely unknown why children differ in such choices and what influences their decisions. Because their A-level grades are used for university admission, it is reasonable to assume that children choose subjects in which they expect to do well or choose the subjects they enjoy, as they are required to focus and put substantial effort in these disciplines during the two A-level years. The focus of the current study is to investigate the extent to which students’ choice to do A-levels and their choice of A-level subjects as well as subsequent achievement can be explained by genetic or environmental influences.

## The current study

The study used a large UK-representative twin sample, the Twins Early Development Study (TEDS)^16^, to investigate the genetic and environmental contributions in choosing to do A-levels and subject choice at age 16, as well as achievement in the chosen subjects at age 18. Based on previous research, we hypothesized that the heritability of school achievement at age 18 would be substantial, and that it would be substantial across the multiple subjects children study at school after compulsory education. We also investigated, for the first time, the extent to which the decision to continue studying at A-level and the students’ subject choice is made on the basis of their genetic propensities. The design also estimates the influence of shared environmental factors that reflect shared school and family influences and non-shared environment such as child-specific school recommendations and parental advice for choosing to do A-levels and for choosing specific subjects.

## Results

### Descriptive statistics

Table 1 presents the proportion of students taking A-level and their subject choices for the whole sample, for boys and girls separately, and for each of the five zygosity groups: MZ males, MZ females, DZ males, DZ females and DZ opposite-sex twin pairs. Using the TEDS data collected at age 18, we show that about 50% of the participants (6613 students from the overall sample of 13,168, of whom 7012 were female and 6156 were male) choose to continue their studies at A-level, which is similar to the UK national average (42% of students in the 16–18 year cohort in England and Wales continue to do A-levels: https://www.gov.uk/government/uploads/system/uploads/attachment_data/file/502158/SFR03_2016__A_level_and_other_level_3_results_in_England_SFR_revised.pdf) . There were significant differences between girls and boys for choosing to do A-levels; 57% of females chose to study at A-level compared to 43% of males. Overall, girls and boys choose STEM subjects in equal proportions (49% girls, 51% boys), although girls much prefer biology (63% girls, 37% boys) and boys much prefer physics (23% girls, 77% boys). Boys slightly prefer mathematics (42% girls, 58% boys); there is little difference in chemistry (48% girls, 52% boys). Girls more often chose humanities subjects (58% girls, 42% boys), especially English (73% girls, 27% boys), second language (68% girls, 32% boys), and psychology (77% girls, 23% boys).

Although there are substantial sex differences in choice of A-level subjects, Table 2 shows that girls and boys do not differ much in their A-level exam results at age 18. Significant sex differences were found only for the overall A-level grade, humanities composite, and psychology; however, these mean differences were not substantial. ANOVA results show that sex and zygosity explain less than 1% of variance in A-level results except for psychology where they explain 5% of the variance. For the subsequent analyses the data were corrected for the small mean sex and zygosity differences, as described in the Methods section.

### Twin analyses

We investigated quantitative and qualitative sex differences using the full sex-limitation model, as described in the Methods section. No significant qualitative sex differences emerged. Although some significant quantitative sex differences emerged for overall A-level grade, mathematics, chemistry, history and humanities, the differences were small. (Full model fit statistics with the nested models are presented in Supplementary Table 1; ACE estimates and confidence intervals for males and females are listed in Supplementary Table 2.). For example, for A-level mean grade, heritability was 52% (95% CI: 0.38; 0.69) for girls and 57% (95% CI 0.36; 0.69) for boys. The largest difference in heritability was for mathematics 70% (95% CI 0.34; 0.77) girls, and 51% (95% CI 0.15; 0.67); the overlapping confidence intervals for these estimates warrant little confidence because the analysis is underpowered in that only 15% of the sample chose mathematics; the sample was then further reduced by comparing gender as well as zygosity (this is evident by the wide confidence intervals around estimates when calculated for males and females separately). For these reasons, and to increase power in the present analyses, the full sample was used, combining males and females and including opposite-sex pairs.

**Figure 1:**
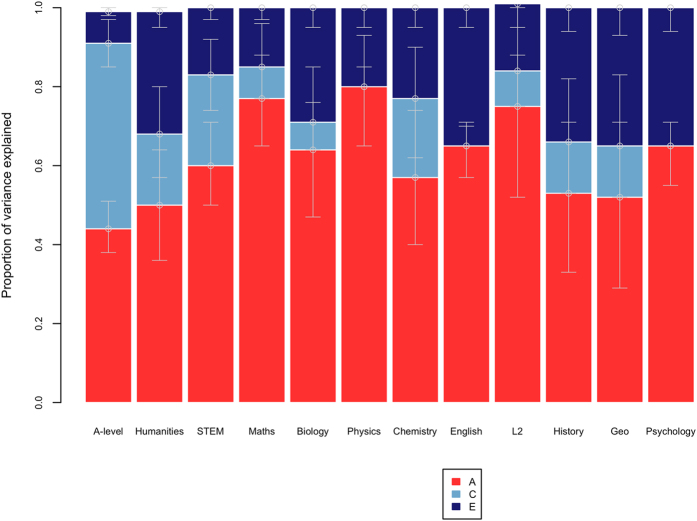
Genetic and environmental estimates for A-level choice and choice of A-level subjects. Liability threhold model-fitting results (error bars representing the 95% confidence intervals). A = additive genetic, C = shared environmental and E = non-shared environmental components of variance. STEM = science, technology, engineering and mathematics, Geo = geography, L2= second language

We used the liability threshold model to calculate ACE estimates for choosing to study at A-level and A-level subject choice, as described in the Methods section. As illustrated in Fig. 1, choosing to do A-levels was moderately heritable (44%) and the influence of shared environment was just as large (47%). In contrast, the subjects students chose at A-level were more heritable (50–80%) and much less influenced by shared environment (0–23%). (Twin tetrachoric correlations and full-model fit statistics with confidence intervals are presented in Supplementary Table 3.).

**Figure 2:**
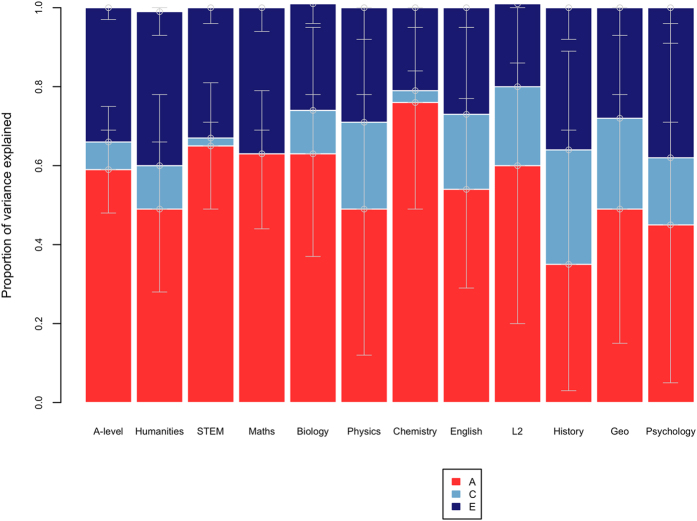
Genetic and environmental estimates for A-level exam results: univariate model-fitting results (error bars representing the 95% confidence intervals). A = additive genetic, C = shared environmental and E = non-shared environmental components of variance. STEM = science, technology, engineering and mathematics, Geo = geography, L2 = second language.

Figure 2 presents ACE estimates for academic achievement at age 18. A-level mean performance was highly heritable (59%) with only a small proportion of the variance explained by shared environmental factors (7%). Heritability was non-significantly lower for the humanities composite (49%) compared to STEM (65%). Although heritabilities differed across subjects from 35% for history to 76% for chemistry, the sample size was too small to provide adequate power to detect such differences, which can be seen in the estimates’ overlapping confidence intervals. (Full-model fit statistics with confidence intervals are presented in Supplementary Table 4.).

## Discussion

These results show, for the first time, that genetic factors influence academic choice, not just achievement. Whether or not 16-year-olds choose to continue their studies at A-level in preparation for university is influenced in equal measure by genetic (44%) and shared environmental factors (47%). Choosing specific A-level subjects is more heritable (50% for humanities, 60% for STEM) and less influenced by shared environment (18% for humanities, 23% for STEM). Genetic factors affect subject choice across a wide range of school subjects, including second language learning, mathematics and psychology. We could not repeat the analyses across all A-level subjects because some subjects were chosen by very few students. For example, it would have been interesting to study the etiology of subject choice for more art-related subjects, such as art, drama and music, but it was not possible in the present study because of limited power.

How can DNA differences affect choice? Two obvious possibilities are previous achievement and ability. That is, it seems reasonable to expect that students make A-level choices in part on the basis of previous educational achievement, which is substantially heritable. It is also possible that general intelligence, which is also substantially heritable, contributes to these choices independently from previous achievement. We are currently investigating the role of earlier achievement and ability, but we are especially interested in the less obvious possibility that choice is governed by appetites as well as by achievement and ability. In other words, it seems likely that students choose subjects they enjoy, and this could be a cause rather than just an effect of their previous achievement. Our ongoing research capitalizes on the longitudinal data available from this sample to explore the motivational antecedents of choice. Our future research plans also include using all the data collected in TEDS longitudinally to study the early and concurrent predictors and correlates of educational achievement and subject choice at A-levels.

Another noteworthy aspect of the results in relation to choice is the substantial influence of shared environment on choosing to do two years of A-level studies. We found that nearly half (47%) of the liability to make this choice can be attributed to shared environment. Although it does not seem surprising that parents and teachers influence both members of a twin pair to make similar choices about whether to do A-levels, this finding is noteworthy because, despite its reasonableness, it is rare to find such a major role for shared environment for other traits. It is possible that teachers and parents encourage both children in a twin pair to continue their studies at A-levels, but that specific career advice is more personalized. As noted above, shared environment has only half as much impact on choice of A-level subjects (23% for STEM; 18% for humanities), and it has even less effect on A-level grades (2% for STEM; 11% for humanities). We are using the longitudinal data from TEDS to investigate the specific aspects of the shared environment that influence A-level choice and to explore why these same environmental factors have less of an influence on specific subject choice and achievement.

Finding substantial heritability for A-level exam scores at age 18 (65% for STEM; 49% for humanities) is consistent with our earlier research showing that educational achievement is highly heritable across compulsory education^5^,^6^,^11^,^12^. Nonetheless, this finding is remarkable because only half the population chooses to do A-levels, both in TEDS and in the UK (https://www.gov.uk/government/uploads/system/uploads/attachment_data/file/207749/Main_text_-_SFR19_2013.pdf). This self-selection leads to a restriction of range of ability among these university-bound students. Despite this restriction of range, DNA differences continue to differentiate performance on A-level exams to a similar extent as achievement during earlier years when education was compulsory for all children.

Although it has been said many times, it is worth reiterating that heritability does not imply immutability. Heritability describes the extent to which phenotypic differences between individuals can be explained by genetic differences in a particular population with that population’s mix of genetic and environmental influences at that time. Therefore, the findings of the current study may not generalize to other populations. In other words, heritability describes what is, not what could be. High heritability of educational achievement does not doom attempts to have all children reach a minimal level of literacy or numeracy. In the same way, finding that shared environmental influence is modest for A-level achievement does not mean that schools or teachers are unimportant. Instead, these results indicate that children’s educational potential could be maximized if environments were more personalized and suited to their specific needs. We hope that the findings of the present study will lead to further research in other populations to advance understanding of educational choices and achievement throughout school years and beyond.

Our findings imply that inherited differences in DNA sequence are associated with academic choice and achievement. Nothing would advance research in this area more than identifying the specific DNA sequences responsible for heritability. This is beginning to happen, for example, for educational attainment^17^ and for general intelligence^18^. However, the main finding to date across the life sciences is that the heritability of complex traits and common disorders is due to many, perhaps thousands, of DNA differences, each of very small effect size. Indeed, the largest effect size for educational attainment (years of schooling) is an association that accounts for a mere 0.02% of the variance in a genome-wide association meta-analysis with a sample of 120,000^17^. Rather than focusing on a handful of such DNA differences that reach genome-wide statistical significance, researchers are beginning to use polygenic scores that aggregate thousands of DNA differences^19^. Even so, the missing heritability gap is large; for example, for educational attainment, a polygenic score obtained from a recent genome-wide association meta-analysis of educational attainment with nearly 300,000 adults accounted for about 5% of the variability for educational attainment in independent samples, even though this variable is about 50% heritable^20^. Nonetheless, we have shown in our sample that this polygenic score for educational attainment in adults is significantly associated with educational achievement and general intelligence in 16-year-olds^21^. In our future research, we will use this polygenic score and other polygenic scores (together with the SNP-based methods) to investigate academic choice and achievement at A-levels. Polygenic scores are needed that are derived from bigger and better genome-wide association studies – that is, with bigger samples that can detect even smaller effects and with better measures of educational achievement rather than the proxy measure of educational attainment.

Finding substantial genetic influence on choice as well as achievement supports a genetic way of thinking about education in which individuals actively choose and create educational experiences on the basis of their genetic propensities, called *genotype-environment correlation*^22^. This active view of education (‘leading out’) contrasts with the traditional passive model of instruction (‘shoving in’). Giving children a more active choice in their curricula would allow children to become more active participants in their education rather than passive receivers of instruction. Finding genetic influence on choice as well as achievement does not dictate any specific policies, but it supports educational trends away from a one-size-fits-all curriculum towards providing more opportunities, choice and personalized learning, helping each child to reach their maximum potential.

## Method

### Participants

The sample was drawn from the Twins Early Developmental Study (TEDS), a representative sample of twins born in England and Wales between 1994 and 1996. Of the 16,000 twin pairs originally recruited, over 10,000 remain actively involved in TEDS. Their recruitment and representativeness has been described in detail elsewhere^5^,^23^. The present study included all individuals with educational achievement data available at 18. Participants with severe medical or psychiatric problems or whose mothers had severe medical complications during pregnancy were excluded from the analysis. We also excluded participants with unknown zygosity. Zygosity was assessed by a parent-reported questionnaire of physical similarity, which is over 95% accurate when compared to DNA testing^24^. For cases where zygosity was unclear from this questionnaire, DNA testing was conducted. After exclusions, the total number of individuals for whom data at 18 were available was 13,226 individuals (6584 twin pairs), of whom 2318 were monozygotic (MZ) twin pairs, 2146 were dizygotic same-sex pairs (DZss) and 2120 were dizygotic opposite-sex (DZos) pairs. A-level exam achievement results were available for half of the participants (the proportion of participants who took the A-level exams): 3308 twin pairs of which 1178 were MZ twin pairs, 1067 were DZ same-sex twin pairs, and 1063 were DZ opposite-sex twin pairs.

In the twin method, DZ twin pairs are needed to delineate genetic and environmental contributions to a trait, with same-sex DZ twins most often used because they provide a more appropriate control for MZ twin pairs, who are always the same sex^4^,^25^. When data are available from opposite-sex twin pairs, sex differences in the etiology of individual differences can also be explored. Sex limitation results are reported in the Results section. Because little evidence was found for significant sex differences for the achievement data and to increase power, we used the full sample, including opposite-sex twin pairs.

### Measures

The TEDS sample has now completed compulsory education. In England and Wales, compulsory education ends with the General Certificate of Secondary Education (GCSE), a standardized examination typically taken at the age of 16. Completion of GCSE examinations marks a unique stage for pupils who are now, for the first time, free to choose whether to leave formal education or to continue their studies to complete further education (FE). In the UK, FE refers to courses offered in separate FE colleges or more commonly, available within the sixth-form part of a school, which are distinct from the undergraduate and graduate degrees typically offered at universities (http://www.cambridgeassessment.org.uk/Images/140668-popularity-of-a-level-subjects-among-uk-university-students.pdf). These FE qualifications are commonly taken over a two-year period, with official examinations held at the end of each year, leading to a formal qualification known as the General Certificate of Education Advanced level, or A-level, which is the focus of the present study. Alternative qualifications including the International Baccalaureate, NVQ (National Vocational Qualification) and BTEC (Business and Technology Education Council) are also considered FE but were not analyzed in the present study (https://www.studential.com/further-education/vocational-qualifications).

Unlike in previous school years, at A-level pupils are free to choose all of their courses from over 80 different subjects, typically choosing three to four subjects studied during the two-year period (http://www.cambridgeassessment.org.uk/Images/140668-popularity-of-a-level-subjects-among-uk-university-students.pdf). Grades achieved in both exams (GCSE and A-level) are converted into a points-based system (https://www.ucas.com/ucas/undergraduate/getting-started/entry-requirements/tariff), which is evaluated by the student’s chosen university along with previous school performance and teacher-predicted results, as criteria for university entry. However, some universities evaluate specific grades achieved, not just achievement based on the overall points-based system. A detailed description of the UK education system can be found on UK Department of Education website (https://www.gov.uk/government/uploads/system/uploads/attachment_data/file/219167/v01-2012ukes.pdf).

A questionnaire, designed to obtain A-level and other post-16 qualifications as well as work destinations, was sent to all TEDS families at the end of the academic school year when twins reached age 18. The full questionnaire was completed either by twins themselves or by their parents. We have previously shown that self-reported exam results are accurate^12^. For GCSE (General Certificate of Secondary Education) exam results that children take at the age of 16 the grades were verified using the National Pupil Database (NPD; https://www.gov.uk/government/uploads/system/uploads/attachment_data/file/251184/SFR40_2013_FINALv2.pdf) using the sample of 7367 twins, yielding a correlation of 0.99 for mathematics, 0.98 for English and >0.95 for all the sciences.

A-level examination grades (ranging from A* to E) were obtained for each twin and were coded from 6(A*) to 1(E) to ensure equivalent numerical comparisons. Because no subjects at A-level are compulsory and the range of subjects chosen is so wide, the sample sizes were too small to provide adequate power for analyses of separate subjects except for biology, chemistry, physics, history, geography and psychology. For this reason and to increase power generally, we created a composite STEM variable (science, technology, engineering and mathematics), which was derived as a mean grade of all sciences (mean of science, biology, chemistry and physics grades), technology (mean of technology and information communications technology grades), engineering (mean of engineering and mechanical engineering grades), and mathematics (mean of any core mathematics and further mathematics grades) courses. Composites were also created for English (mean of any English language and English literature grades), second language (mean of any second language course grade), and humanities (mean of history, religious studies, media studies and geography grades). An A-level mean grade, computed as the average grade achieved across all subjects in the dataset, was also created to ensure even those subjects with sample sizes too small to be considered separately were included in the analysis. In order to assess individual differences in subject choice we created categorical variables indicating whether or not pupils chose to take the individual or composite subjects described above. Finally, we created a categorical A-level choice variable to indicate whether or not participants chose to do their A-levels.

### Analyses

The data were described in terms of means and variance comparing boys with girls and MZ and DZ twins. Analysis of variance (ANOVA) was then used to explore sex and zygosity differences in means and variances and their interaction, for A-level grades. For subsequent analyses the achievement scores were corrected for small age and sex differences using the regression method because MZ twins are always the same sex, along with the mean effect of age, which is perfectly correlated across pairs, both factors which if uncorrected would inflate estimates of shared environmental influence^26^. Standardized age and sex corrected residuals were used for all subsequent analyses. Finally, prior to conducting twin analyses, the data were corrected for normality using the rank-based van der Waerden transformation^27^,^28^. Corrections were performed because achievement data were slightly positively skewed, showing a ceiling effect similar to data achieved from UK national statistics (https://www.gov.uk/government/uploads/system/uploads/attachment_data/file/365986/SFR42_2014_provisional__A_level_and_level_3_SFR.pdf).

### Twin analyses

In order to investigate the relative genetic and environmental contribution to individual differences in educational achievement, we used the twin design, a quantitative genetic method which exploits the known coefficients of relatedness between identical (MZ) and non-identical (DZ) twins, to apportion phenotypic variance into additive genetic (A), shared environmental (C) and non-shared or unique environmental (E) components. Genetic effects are perfectly correlated for MZ twin pairs who are 100% genetically similar compared to DZ twin pairs who, like non-twin siblings, share 50% of the segregating genes. Shared environmental effects are perfectly correlated for MZ and DZ twin pairs reared together while non-shared environmental effects are uncorrelated for members of a twin pair and do not contribute to similarities between twins. Based on these known relations and the standard quantitative genetic model (Falconer’s formula), heritability (A) can be roughly estimated by doubling the difference between MZ and DZ twin correlations. The residual familial resemblance not explained by heritability is accounted for by the C component, calculated by subtracting the heritability estimate from the MZ correlation. The E component represents the remaining variance and measurement error and is calculated by deducting the A and C components from unity, as the total variance cannot exceed 100%^4^,^25^.

The ACE parameters can be estimated more accurately using structural equation model fitting with maximum-likelihood estimation, which also provides 95% confidence intervals and formal model fit statistics. The structural equation modeling program OpenMx was used for all model fitting analyses^29^.

Power was calculated using Genepi Twin Power calculator^30^,^31^, which shows that the analyses had over 80% power for both the subject choice and achievement variables. The analyses had less than 80% power to detect C in specific subject achievement grades of second language, geography and psychology as is evident from the large confidence intervals around the estimates, but were reported for completeness.

### Sex-limitation model

When data are available for both same sex DZ twin pairs and opposite-sex DZ twins, the standard univariate ACE model can be extended to a sex-limitation model to test the differences in the etiology of the trait of interest by comparing twin correlations across five zygosity groups: MZ males, MZ females, DZ males, DZ females and DZ opposite-sex twin pairs^4^,^32^. Quantitative sex differences refer to sex differences in the magnitude of ACE estimates. Qualitative sex differences test whether there are different genetic or different environmental factors influencing boys and girls separately, which is largely based on whether DZ same-sex twin correlations are higher than DZ opposite-sex correlations^32^.

The sex-limitation model was analyzed using the structural equation program OpenMx by fitting a series of nested models and testing the relative fit of the models^29^. In the full model, all parameters are allowed to vary across all five zygosity groups (genetic correlation between DZos, ACE estimates, variances, ACE estimates, DZss and DZos variances and correlations). To test for qualitative sex differences, the genetic or shared environmental correlation is constrained to expected values (1.0 or 0.5 respectively), while other estimates are allowed to vary in the model. Quantitative genetic differences are tested by a reduced model in which ACE estimates are equated for males and females and the DZos genetic correlation is constrained to 0.5. The sex-limitation model is described in more detail elsewhere^4^,^6^,^32^.

### Liability threshold model

Because subject choice was measured as a dichotomous trait (choosing a subject or not), twin resemblance was assessed by concordances between MZ and DZ twins by comparing the twin who took an A-level course, to their co-twin. Concordance represents an index of risk, often encountered when assessing the presence or absence of a disease; but is used in the present study as the presence or absence of subject choice^4^,^25^. Analyses of categorical twin data assume that observed categories represent an imprecise measurement of an underlying normal distribution of liability^25^. The degree of agreement between MZ twin pairs who are genetically 100% similar is then compared to the degree of agreement between DZ twin pairs, who share 50% of their segregating genes on average using the correlation of liability (tetrachoric correlation). The liability threshold model is described in detail elsewhere^25^. The structural equation program OpenMX was used for the liability threshold model^29^.

## Additional Information

**How to cite this article**: Rimfeld, K. *et al.* Genetics affects choice of academic subjects as well as achievement. *Sci. Rep.* **6**, 26373; doi: 10.1038/srep26373 (2016).

## Supplementary Material

Supplementary Information

## Figures and Tables

**Table 1:** Proportion of the sample choosing to progress to A-level and proportion of participants choosing an A-level subject.

Subject	N*	Male	Female	*X^2^*	MZm	DZm	MZf	DZf	DZos
A-level choice	6613	2826	3787	40.60**	928	934	1428	1197	2126
	(50%)	(43%)	(57%)		(14%)	(14%)	(22%)	(18%)	(32%)
Humanities choice	2561	1068	1493	12.57**	341	345	573	452	850
	(19%)	(42%)	(58%)		(13%)	(14%)	(22%)	(18%)	(33%)
STEM choice	3417	1740	1677	18.57**	573	584	660	539	1061
	(26%)	(51%)	(49%)		(17%)	(17%)	(19%)	(16%)	(31%)
Mathematics choice	1988	1147	841	66.93**	370	371	344	260	643
	(15%)	(58%)	(42%)		(18%)	(19%)	(17%)	(13%)	(32%)
Biology choice	1634	603	1031	36.53**	204	213	374	352	491
	(12%)	(37%)	(63%)		(13%)	(13%)	(23%)	(22%)	(30%)
Physics choice	846	652	194	188.94**	212	220	79	67	268
	(6%)	(77%)	(23%)		(25%)	(26%)	(9%)	(8%)	(32%)
Chemistry choice	1276	608	668	0.73	214	204	236	231	391
	(10%)	(48%)	(52%)		(17%)	(16%)	(19%)	(18%)	(31%)
English composite choice	1807	490	1317	174.43**	164	155	471	414	603
	(14%)	(27%)	(73%)		(9%)	(9%)	(26%)	(23%)	(33%)
Second language choice	544	174	370	28.55**	48	55	166	111	164
	(4%)	(32%)	(68%)		(8%)	(10%)	(31%)	(20%)	(30%)
History choice	1291	571	720	4.54*	178	169	288	211	445
	(10%)	(44%)	(56%)		(14%)	(13%)	(22%)	(16%)	(35%)
Geography choice	1032	466	566	0.01	146	159	217	92	325
	(8%)	(55%)	(45%)		(14%)	(15%)	(21%)	(18%)	(32%)
Psychology choice	1222	285	937	139.37**	107	94	355	267	399
	(9%)	(23%)	(77%)		(9%)	(8%)	(29%)	(22%)	(33%)
Total	13, 168								

N = sample size after exclusions (individuals), proportions of across gender and zygosity groups reported as a proportion of students who chose the subject; MZ = monozygotic; DZ = dizygotic; m = male; f = female; os = opposite sex; *X*^2^ = Chi-square results comparing choice between males and females (one randomly selected twin per pair); *p < 0.05; **p < 0.01.

**Table 2:** Mean scores and (standard deviations) for A-level exam results. Scores for subject means have a maximum grade of 6 and a minimum of 1, representing grades A* to E.

Subject	N	Whole sample	Male	Female	MZm	DZm	MZf	DZf	DZos	Sex	Zyg	Sex x Zyg	R^2^
A-level mean grade	3053	3.90	3.85	3.94	3.82	3.84	3.97	3.89	3.93	4.87*	0.04	1.62	<0.01
		(1.16)	(1.20)	(1.13)	(1.24)	(1.23)	(1.10)	(1.19)	(1.12)				
Humanities mean grade	1280	4.00	3.90	4.07	3.82	3.99	4.11	4.03	3.99	6.84**	0.01	1.91	<0.01
		(1.14)	(1.18)	(1.10)	(1.18)	(1.20)	(1.12)	(1.10)	(1.12)				
STEM mean grade	1723	3.89	3.85	3.92	3.80	3.84	3.92	3.9	3.93	1.01	0.27	0.57	<0.01
		(1.31)	(1.32)	(1.31)	(1.36)	(1.35)	(1.28)	(1.38)	(1.25)				
Mathematics mean grade	1012	4.34	4.27	4.43	4.20	4.27	4.39	4.50	4.37	3.63	0.84	1.33	<0.01
		(1.28)	(1.33)	(1.20)	(1.43)	(1.35)	(1.25)	(1.16)	(1.19)				
Biology grade	812	3.95	3.91	3.98	3.74	4.11	3.87	4.02	3.98	0.53	3.45	1.15	<0.01
		(1.39)	(1.34)	(1.42)	(1.30)	(1.33)	(1.36)	(1.48)	(1.40)				
Physics grade	443	3.97	3.96	4.20	4.07	3.79	4.09	3.79	4.06	0.15	1.20	0.97	<0.01
		(1.38)	(1.38)	1.28	(1.32)	(1.45)	(1.36)	(1.60)	(1.33)				
Chemistry grade	646	4.13	4.05	4.20	3.89	4.23	4.21	4.22	4.12	2.06	1.34	1.33	<0.01
		(1.30)	(1.32)	(1.28)	(1.38)	(1.37)	(1.27)	(1.27)	(1.26)				
English composite grade	904	4.01	4.09	3.98	4.01	4.16	4.08	3.91	3.98	1.50	0.91	0.90	<0.01
		(1.19)	(1.24)	(1.17)	(1.22)	(1.27)	(1.19)	(1.21)	(1.15)				
Second language mean grade	275	4.11	4.15	4.09	4.33	3.90	4.11	4.30	3.96	0.19	0.51	1.20	<0.01
		(1.14)	(1.21)	(1.11)	(1.27)	(1.20)	(0.94)	(1.13)	(1.27)				
History grade	677	4.11	4.06	4.16	4.07	4.1	4.12	4.18	4.1	1.15	0.04	0.13	<0.01
		(1.17)	(1.23)	(1.12)	(1.19)	(1.25)	(1.19)	(1.11)	(1.15)				
Geography grade	496	4	3.91	4.08	3.79	3.97	4.25	3.93	3.99	2.60	0.61	1.97	<0.01
		(1.15)	(1.19)	(1.1)	(1.22)	(1.20)	(1.09)	(1.16)	(1.10)				
Psychology grade	600	3.66	3.31	3.77	3.52	3.45	3.94	3.78	3.44	15.01**	7.07**	4.50**	0.05
		(1.25)	(1.14)	(1.27)	(1.13)	(1.11)	(1.18)	(1.25)	(1.33)				

N = sample size after exclusions; MZ = monozygotic; DZ = dizygotic; m = male; f = female; os = opposite sex. ANOVA analyses (one randomly-selected twin per pair) tested the effect of sex and zygosity: results  = F statistic; *p < 0.05; **p < 0.01; R^2^ = proportion of variance explained by sex, zygosity and their interaction.
