# Supplementary material for: Genetics affects choice of academic subjects as well as achievement
Source: Sci Rep. 2016 Jun 16;6:26373. doi: 10.1038/srep26373 (PMC4910524; doi:10.1038/srep26373)
Supplement: Supplementary Information [file srep26373-s1.pdf]

Supplementary Information

Genetics affects choice of academic subjects as well as achievement

Kaili Rimfeld, Ziada Ayorech, Philip S. Dale, Yulia Kovas and Robert Plomin

Tables

Supplementary Table 1. Sex limitation model fitting sub-model comparisons

Supplementary Table 2. Sex limitation model fitting results, showing A,C,E, estimates separately for males and females. A-additive genetic; C- shared environmental; E- non-shared environmental proportions of the variance (95% confidence intervals)

Supplementary Table 3. Model fitting results for liability threshold analyses for A-level choice with twin tetrachoric correlations (N of twin pairs). A-additive genetic; C- shared environmental; E- non-shared environmental proportions of the variance (95% confidence intervals)

Supplementary Table 4. Model fitting results for univariate analyses for A-level exam achievement with twin intraclass correlations (N of complete pairs). A-additive genetic; C- shared environmental; E- non-shared environmental proportions of the variance (95% confidence intervals)

Supplementary Table 1. Sex limitation model fitting sub-model comparisons. All comparisons are made with the full sex-limitation model as explained in the Methods. ep= estimated parameters; -2LL= -2 log-likelihood; df= degrees of freedom; AIC= Akaike's information criteria; diffLL= change in log-likelihood; diffdf= change in degrees of freedom.

A-level mean grade

**Qualitative genetic differences**							
Model	ep	-2LL	df	AIC	diffLL	diffdf	p
FullHetACE	9	16545.85	6067	4411.85	-	-	-
HetACE	8	16546.29	6068	4410.29	0.45	1	0.5
**Qualitative environmental differences**							
Model	ep	-2LL	df	AIC	diffLL	diffdf	p
cFullHetACE	9	16545.85	6067	4411.85	-	-	-
HetACE	8	16546.29	6068	4410.29	0.45	1	0.5
**Quantitative genetic differences**							
Model	ep	-2LL	df	AIC	diffLL	diffdf	p
HetACE	8	16546.29	6068	4410.29	-	-	-
HomACE	5	16561.16	6071	4419.16	14.86	3	0

Humanities mean grade

**Qualitative genetic differences**							
Model	ep	-2LL	df	AIC	diffLL	diffdf	p
FullHetACE	9	7164.26	2586	1992.26	-	-	-
HetACE	8	7164.26	2587	1990.26	0	1	0.97
**Qualitative environmental differences**							
Model	ep	-2LL	df	AIC	diffLL	diffdf	p
cFullHetACE	9	7164.26	2586	1992.26	-	-	-
HetACE	8	7164.26	2587	1990.26	0	1	0.97
**Quantitative genetic differences**							
Model	ep	-2LL	df	AIC	diffLL	diffdf	p
HetACE	8	7164.26	2587	1990.26	-	-	-
HomACE	5	7176.78	2590	1996.78	12.53	3	0.01

STEM mean grade

**Qualitative genetic differences**							
Model	ep	-2LL	df	AIC	diffLL	diffdf	p
FullHetACE	9	9366.22	3399	2568.22	-	-	-
HetACE	8	9366.27	3400	2566.27	0.05	1	0.82
**Qualitative environmental differences**							
Model	ep	-2LL	df	AIC	diffLL	diffdf	p
cFullHetACE	9	9366.22	3399	2568.22	-	-	-
HetACE	8	9366.27	3400	2566.27	0.05	1	0.82
**Quantitative genetic differences**							
Model	ep	-2LL	df	AIC	diffLL	diffdf	p
HetACE	8	9366.27	3400	2566.27	-	-	-
HomACE	5	9372.89	3403	2566.89	6.61	3	0.09

Mathematics mean grade

**Qualitative genetic differences**							
Model	ep	-2LL	df	AIC	diffLL	diffdf	p
FullHetACE	9	5497.6	1993	1511.6	-	-	-
HetACE	8	5497.95	1994	1509.95	0.35	1	0.55
**Qualitative environmental differences**							
Model	ep	-2LL	df	AIC	diffLL	diffdf	p
cFullHetACE	9	5498.73	1993	1512.73	-	-	-
HetACE	8	5497.95	1994	1509.95	-0.79	1	1
**Quantitative genetic differences**							
Model	ep	-2LL	df	AIC	diffLL	diffdf	p
HetACE	8	5497.95	1994	1509.95	-	-	-
HomACE	5	5518.38	1997	1524.38	20.43	3	0

Biology mean grade

**Qualitative genetic differences**							
Model	ep	-2LL	df	AIC	diffLL	diffdf	p
FullHetACE	9	4481.7	1625	1231.7	-	-	-
HetACE	8	4481.91	1626	1229.91	0.21	1	0.64
**Qualitative environmental differences**							
Model	ep	-2LL	df	AIC	diffLL	diffdf	p
cFullHetACE	9	4481.7	1625	1231.7	-	-	-
HetACE	8	4481.91	1626	1229.91	0.21	1	0.64
**Quantitative genetic differences**							
Model	ep	-2LL	df	AIC	diffLL	diffdf	p
HetACE	8	4481.91	1626	1229.91	-	-	-
HomACE	5	4490.56	1629	1232.56	8.65	3	0.03

Physics mean grade

**Qualitative genetic differences**							
Model	ep	-2LL	df	AIC	diffLL	diffdf	p
FullHetACE	9	2319.59	837	645.59	-	-	-
HetACE	8	2319.81	838	643.81	0.22	1	0.64
**Qualitative environmental differences**							
Model	ep	-2LL	df	AIC	diffLL	diffdf	p
cFullHetACE	9	2319.52	837	645.52	-	-	-
HetACE	8	2319.81	838	643.81	0.29	1	0.59
**Quantitative genetic differences**							
Model	ep	-2LL	df	AIC	diffLL	diffdf	p
HetACE	8	2319.81	838	643.81	-	-	-
HomACE	5	2322.17	841	640.17	2.35	3	0.5

Chemistry mean grade

Model	ep	-2LL	df	AIC	diffLL	diffdf	p
**Qualitative genetic differences**							
Model	ep	-2LL	df	AIC	diffLL	diffdf	p
FullHetACE	9	3472.7	1267	938.7	-	-	-
HetACE	8	3472.7	1268	936.7	0	1	1
**Qualitative environmental differences**							
Model	ep	-2LL	df	AIC	diffLL	diffdf	p
cFullHetACE	9	3472.7	1267	938.7	-	-	-
HetACE	8	3472.7	1268	936.7	0	1	1
**Quantitative genetic differences**							
Model	ep	-2LL	df	AIC	diffLL	diffdf	p
HetACE	8	3472.7	1268	936.7	-	-	-
HomACE	5	3483.09	1271	941.09	10.4	3	0.02

English mean grade

Model	ep	-2LL	df	AIC	diffLL	diffdf	p
**Qualitative genetic differences**							
Model	ep	-2LL	df	AIC	diffLL	diffdf	p
FullHetACE	9	4980.84	1809	1362.84	-	-	-
HetACE	8	4981.73	1810	1361.73	0.89	1	0.34
**Qualitative environmental differences**							
Model	ep	-2LL	df	AIC	diffLL	diffdf	p
cFullHetACE	9	4980.77	1809	1362.77	-	-	-
HetACE	8	4981.73	1810	1361.73	0.95	1	0.33
**Quantitative genetic differences**							
Model	ep	-2LL	df	AIC	diffLL	diffdf	p
HetACE	8	4981.73	1810	1361.73	-	-	-
HomACE	5	4984.88	1813	1358.88	3.15	3	0.37

Second language mean grade

**Qualitative genetic differences**							
Model	ep	-2LL	df	AIC	diffLL	diffdf	p
FullHetACE	9	1497.69	544	409.69	-	-	-
HetACE	8	1497.69	545	407.69	0	1	1
**Qualitative environmental differences**							
Model	ep	-2LL	df	AIC	diffLL	diffdf	p
cFullHetACE	9	1497.69	544	409.69	-	-	-
HetACE	8	1497.69	545	407.69	0	1	1
**Quantitative genetic differences**							
Model	ep	-2LL	df	AIC	diffLL	diffdf	p
HetACE	8	1497.69	545	407.69	-	-	-
HomACE	5	1499.21	548	403.21	1.52	3	0.68

History mean grade

**Qualitative genetic differences**							
Model	ep	-2LL	df	AIC	diffLL	diffdf	p
FullHetACE	9	3556.09	1279	998.09	-	-	-
HetACE	8	3556.09	1280	996.09	0	1	1
**Qualitative environmental differences**							
Model	ep	-2LL	df	AIC	diffLL	diffdf	p
cFullHetACE	9	3556.09	1279	998.09	-	-	-
HetACE	8	3556.09	1280	996.09	0	1	1
**Quantitative genetic differences**							
Model	ep	-2LL	df	AIC	diffLL	diffdf	p
HetACE	8	3556.09	1280	996.09	-	-	-
HomACE	5	3567.82	1283	1001.82	11.73	3	0.01

Geography mean grade

**Qualitative genetic differences**							
Model	ep	-2LL	df	AIC	diffLL	diffdf	p
FullHetACE	9	2816.36	1018	780.36	-	-	-
HetACE	8	2816.36	1019	778.36	0	1	1
**Qualitative environmental differences**							
Model	ep	-2LL	df	AIC	diffLL	diffdf	p
cFullHetACE	9	2816.36	1018	780.36	-	-	-
HetACE	8	2816.36	1019	778.36	0	1	1
**Quantitative genetic differences**							
Model	ep	-2LL	df	AIC	diffLL	diffdf	p
HetACE	8	2816.36	1019	778.36	-	-	-
HomACE	5	2820.89	1022	776.89	4.53	3	0.21

Psychology mean grade

**Qualitative genetic differences**							
Model	ep	-2LL	df	AIC	diffLL	diffdf	p
FullHetACE	9	3371.81	1208	955.81	-	-	-
HetACE	8	3371.81	1209	953.81	0	1	1
**Qualitative environmental differences**							
Model	ep	-2LL	df	AIC	diffLL	diffdf	p
cFullHetACE	9	3371.81	1208	955.81	-	-	-
HetACE	8	3371.81	1209	953.81	0	1	1
**Quantitative genetic differences**							
Model	ep	-2LL	df	AIC	diffLL	diffdf	p
HetACE	8	3371.81	1209	953.81	-	-	-
HomACE	5	3376.84	1212	952.84	5.03	3	0.17

Supplementary Table 2. Sex limitation model fitting results, showing A, C, E, estimates separately for males and females. A- additive genetic; C- shared environmental; E- non-shared environmental proportions of the variance (95% confidence intervals)

	Males			Females		
Subject	**A**	**C**	**E**	**A**	**C**	**E**
A-level mean grade	0.57 (0.38-0.69)	0.08 (0-0.24)	0.35 (0.31-0.41)	0.52 (0.36-0.69)	0.15 (0-0.29)	0.33 (0.29-0.37)
Humanities mean grade	0.55 (0.18-0.72)	0.09 (0-0.40)	0.36 (0.28-0.48)	0.45 (0.10-0.65)	0.12 (0-0.43)	0.43 (0.35-0.53)
STEM mean grade	0.60 (0.34-0.69)	0.03 (0-0.25)	0.37 (0.31-0.45	0.61 (0.36-0.76)	0.10 (0-0.33)	0.29 (0.24-0.35)
Mathematics mean grade	0.51 (0.15-0.67)	0.08 (0-0.38)	0.41 (0.33-0.52)	0.70 (0.34-0.77)	0.00 (0-0.33)	0.30 (0.23-0.40)
Biology grade	0.23 (0-0.74)	0.46 (0-0.72)	0.31 (0.21-0.46)	0.65 (0.35-0.80)	0.09 (0-0.36)	0.26 (0.19-0.36)
Physics grade	0.36 (0-0.79)	0.37 (0-0.71)	0.27 (0.19-0.40)	0.45 (0-0.82)	0.21 (0-0.75)	0.34 (0.18-0.70)
Chemistry grade	0.76 (0.40-0.85)	0.03 (0-0.35)	0.21 (0.15-0.43)	0.56 (0.17-0.85)	0.23 (0-0.58)	0.21 (0.15-0.31)
English composite grade	0.38 (0-0.80)	0.33 (0-0.72)	0.30 (0.18-0.51)	0.34 (0.04-0.72)	0.40 (0.02-0.66)	0.26 (0.21-0.34)
Second language mean grade	0.82 (0.04-0.94)	0.06 (0-0.74)	0.12 (0.06-0.37)	0.47 (0-0.84)	0.30 (0-0.74)	0.23 (0.15-0.37)
History grade	0.38 (0-0.67)	0.20 (0-0.55)	0.42 (0.27-0.65)	0.10 (0-0.65)	0.56 (0.04-0.73)	0.34 (0.24-0.47)
Geography grade	0.24 (0-0.78)	0.47 (0-0.74)	0.29 (0.18-0.47)	0.63 (0.21-0.80)	0.09 (0-0.44)	0.28 (0.19-0.44)
Psychology grade	0.00 (0-0.72)	0.73 (0-0.84)	0.27 (0.16-0.48)	0.45 (0.07-0.68)	0.14 (0-0.48)	0.41 (0.30-0.56)

Supplementary Table S3. Model fitting results for liability threshold analyses for A-level choice with twin tetrachoric correlations. A-additive genetic; C- shared environmental; E- non-shared environmental proportions of the variance (95% confidence intervals)

				Twin tetrachoric correlations	
Subject choice	**A**	**C**	**E**	**MZ**	**DZ**
A-level	0.44 (0.38-0.51)	0.47 (0.41-0.53)	0.08 (0.07-0.10)	0.92 (0.90-0.93)	0.69 (0.66-0.72)
Humanities composite	0.50 (0.36-0.64)	0.18 (0.07-0.30)	0.31 (0.27-0.36)	0.69 (0.64-0.74)	0.44 (0.38-0.49)
STEM composite	0.60 (0.50-0.71)	0.23 (0.14-0.32)	0.17 (0.14-0.32)	0.83 (0.80-0.86)	0.65 (0.49-0.57)
Mathematics	0.77 (0.65-0.88)	0.08 (0-0.19)	0.15 (0.12-0.18)	0.86 (0.82-0.89)	0.47 (0.41-0.52)
Biology	0.64 (0.47-0.76)	0.07 (0-0.21)	0.29 (0.24-0.35)	0.71 (0.67-0.75)	0.39 (0.34-0.43)
Physics	0.80 (0.65-0.85)	0.00 (0-0.13)	0.20 (0.15-0.26)	0.81 (0.74-0.86)	0.38 (0.29-0.46)
Chemistry	0.57 (0.40-0.74)	0.20 (0.05-0.33)	0.23 (0.18-0.29)	0.77 (0.71-0.82)	0.48 (0.41-0.54)
English composite	0.65 (0.57-0.70)	0.00 (0-0.06)	0.35 (0.30-0.41)	0.67 (0.61-0.73)	0.27 (0.20-0.33)
Second language	0.75 (0.52-0.88)	0.09 (0-0.29)	0.17 (0.11-0.23)	0.84 (0.78-0.89)	0.45 (0.35-0.55)
History	0.53 (0.33-0.71)	0.13 (0-0.29)	0.34 (0.28-0.41)	0.66 (0.59-0.73)	0.40 (0.33-0.47)
Geography	0.52 (0.29-0.71)	0.13 (0-0.31)	0.35 (0.28-0.43)	0.65 (0.57-0.72)	0.40 (0.32-0.47)
Psychology	0.65 (0.55-0.71)	0 (0-0)	0.35 (0.29-0.41)	0.69 (0.62-0.75)	0.26 (0.17-0.34)

Supplementary Table S4. Model fitting results for univariate analyses for A-level exam achievement with twin intraclass correlations (N of complete pairs). A-additive genetic; C- shared environmental; E- non-shared environmental proportions of the variance (95% confidence intervals)

				Twin intraclass correlations	
Subject	**A**	**C**	**E**	**MZ**	**DZ**
A-level mean grade	0.59 (0.48-0.69)	0.07 (0-0.16)	0.34 (0.31-0.37)	0.64 (1076) (0.60-0.68)	0.36 (1972) (0.32-0.41)
Humanities mean grade	0.49 (0.28-0.66)	0.11 (0-0.29)	0.39 (0.33-0.47)	0.61 (462) (0.53-0.68)	0.36 (815) (0.26-0.45)
STEM mean grade	0.65 (0.49-0.71)	0.02 (0-0.16)	0.33 (0.29-0.38)	0.65 (616) (0.59-0.70)	0.32 (1106) (0.25-0.40)
Mathematics mean grade	0.63 (0.44-0.69)	0.00 (0-0.16)	0.37 (0.31-0.44)	0.63 (364) (0.55-0.70)	0.24 (648) (0.12-0.36)
Biology grade	0.63 (0.37-0.78)	0.11 (0-0.32)	0.27 (0.22-0.35)	0.71 (279) (0.62-0.78)	0.43 (533) (0.29-0.55)
Physics grade	0.49 (0.12-0.78)	0.22 (0-0.54)	0.29 (0.21-0.40)	0.71 (151) (0.57-0.80)	0.51 (292) (0.28-0.68)
Chemistry grade	0.76 (0.49-0.84)	0.03 (0-0.28)	0.21 (0.16-0.28)	0.79 (225) (0.71-0.85)	0.38 (421) (0.22-0.52)
English composite grade	0.54 (0.29-0.77)	0.19 (0-0.41)	0.27 (0.22-0.34)	0.71 (312) (0.62-0.78)	0.45 (592) (0.31-0.57)
Second language mean grade	0.60 (0.20-0.86)	0.20 (0-0.55)	0.21 (0.21-0.31)	0.64 (110) (0.47-0.77)	0.47 (164) (0.15-0.70)
History grade	0.35 (0.03-0.69)	0.29 (0-0.54)	0.36 (0.28-0.48)	0.64 (235) (0.51-0.74)	0.46 (440) (0.29-0.60)
Geography grade	0.49 (0.15-0.78)	0.23 (0-0.51)	0.28 (0.21-0.40)	0.74 (182) (0.61-0.83)	0.49 (312) (0.30-0.64)
Psychology grade	0.45 (0.05-0.71)	0.17 (0-0.51)	0.38 (0.29-0.51)	0.58 (221) (0.43-0.69)	0.39 (379) (0.17-0.57)
